# Genomes to Fields 2022 Maize genotype by Environment Prediction Competition

**DOI:** 10.1186/s13104-023-06421-z

**Published:** 2023-07-17

**Authors:** Dayane Cristina Lima, Jacob D. Washburn, José Ignacio Varela, Qiuyue Chen, Joseph L. Gage, Maria Cinta Romay, James Holland, David Ertl, Marco Lopez-Cruz, Fernando M. Aguate, Gustavo de los Campos, Shawn Kaeppler, Timothy Beissinger, Martin Bohn, Edward Buckler, Jode Edwards, Sherry Flint-Garcia, Michael A. Gore, Candice N. Hirsch, Joseph E. Knoll, John McKay, Richard Minyo, Seth C. Murray, Osler A. Ortez, James C. Schnable, Rajandeep S. Sekhon, Maninder P. Singh, Erin E. Sparks, Addie Thompson, Mitchell Tuinstra, Jason Wallace, Teclemariam Weldekidan, Wenwei Xu, Natalia de Leon

**Affiliations:** 1 Department of Agronomy, University of Wisconsin – Madison, Madison, WI 53706 USA; 2 USDA-ARS Plant Genetics Research Unit, 205 Curtis Hall, Columbia, MO 65211 USA; 3 Department of Crop and Soil Sciences, North Carolina State University, Raleigh, NC 27695 USA; 4 Institute for Genomic Diversity, Cornell University, Ithaca, NY 14853 USA; 5 USDA-ARS Plant Science Research Unit, Raleigh, NC 27606 USA; 6 Iowa Corn Promotion Board, Johnston, IA 50131 USA; 7 Department of Epidemiology and Biostatistics, Institute for Quantitative Health Science and Engineering, Michigan State University, East Lansing, MI 48824 USA; 8 Department of Plant, Soil and Microbial Sciences, Department of Epidemiology and Biostatistics, Institute for Quantitative Health Science and Engineering, Michigan State University, East Lansing, MI 48824 USA; 9 Department of Crop Science, Center for Integrated Breeding Research, University of Göttingen, Carl-Sprengel-Weg 1, 37075 Göttingen, Germany; 10 University of Illinois at Urbana-Champaign, Urbana, IL 61801 USA; 11 USDA-ARS and Cornell University, Ithaca, NY 14853 USA; 12 USDA ARS CICGRU, 716 Farmhouse Ln, Ames, IA 50011-1051 USA; 13 Plant Breeding and Genetics Section, School of Integrative Plant Science, Cornell University, Ithaca, NY 14853 USA; 14 Department of Agronomy and Plant Genetics, University of Minnesota, St Paul, MN 55108 USA; 15 USDA-ARS Crop Genetics and Breeding Research Unit, Tifton, GA 31793 USA; 16 Department of Agricultural Biology, Colorado State University, Fort Collins, CO 80523 USA; 17 Department of Horticulture and Crop Science, College of Food, Agricultural, and Environmental Sciences, Ohio State University, Wooster, OH 44691 USA; 18 Department of Soil and Crop Sciences, Texas A&M University, College Station, TX 77843 USA; 19 Department of Horticulture and Crop Science, Ohio State University, Columbus, OH 43210 USA; 20 Department of Agronomy and Horticulture, University of Nebraska-Lincoln, Lincoln, NE 68588 USA; 21 Department of Genetics and Biochemistry, Clemson University, Clemson, SC 29634 USA; 22 Department of Plant, Soil and Microbial Sciences, Michigan State University, East Lansing, MI 48824 USA; 23 Department of Plant and Soil Sciences, University of Delaware, Newark, DE 19716 USA; 24 Department of Agronomy, Purdue University, West Lafayette, IN 49707 USA; 25 Department of Crop & Soil Sciences, University of Georgia, Athens, GA 30602 USA; 26 Texas A&M University, College Station, TX 77843 USA

**Keywords:** Grain yield, Maize, Root mean squared error

## Abstract

**Objectives:**

The Genomes to Fields (G2F) 2022 Maize Genotype by Environment (GxE) Prediction Competition aimed to develop models for predicting grain yield for the 2022 Maize GxE project field trials, leveraging the datasets previously generated by this project and other publicly available data.

**Data description:**

This resource used data from the Maize GxE project within the G2F Initiative [1]. The dataset included phenotypic and genotypic data of the hybrids evaluated in 45 locations from 2014 to 2022. Also, soil, weather, environmental covariates data and metadata information for all environments (combination of year and location). Competitors also had access to ReadMe files which described all the files provided. The Maize GxE is a collaborative project and all the data generated becomes publicly available [2]. The dataset used in the 2022 Prediction Competition was curated and lightly filtered for quality and to ensure naming uniformity across years.

## Objective

The Maize GxE project is a collaborative effort that involves researchers from diverse areas of study. The datasets collected by the project are some of the largest public data of their kind and are therefore of broad interest to communities from genetics to agronomy to computer science and beyond. The competition was organized to connect these communities and others with interest in dissecting and exploring genotypic, environmental, and GxE information to predict hybrid maize performance in different environments across the US. The competition started on November 15, 2022, and ended on January 15, 2023. All the participants had access to the same curated data set, containing information collected on over 180,000 maize field plots and involving 4,683 hybrids. Participants were asked to create predictive models for maize grain yield for the 2022 Maize GxE project field trials, utilizing the existing Maize GxE project dataset and any other publicly available data. The trait of interest was grain yield, and the competitors were asked to submit absolute grain yield (Mg ha^− 1^) adjusted to 15.5% moisture for each hybrid in each location where data had been collected during the 2022 field season. The winner of the competition was the model with the lowest average root mean squared error (RMSE) across locations when compared with the actual yield data obtained in 2022.

## Data description

The Prediction Competition data are publicly available via CyVerse/iPlant. This dataset contains training and testing set data and has been structured according to the specifications outlined in Table 1.

- **Training_data**: includes phenotypic, genotypic, soil, weather (downloaded from https://power.larc.nasa.gov), environmental covariate data, and metadata information from 2014 to 2021 for use in developing and training models.
- **Testing_data**: includes genotypic, soil, weather, environmental covariate data, and metadata information for 2022 locations. Also, a submission template that contains the environments and hybrids that participants used to submit yield predictions.

Maize is cultivated as a hybrid crop, typically resulting from the cross of two inbred parents. Consequently, both the phenotypic data in the training and testing sets exhibit hybrid information. The genotypic data includes hybrid information generated in-silico from inbred genotypic data.

**Table 1:** Overview of Genomes to Fields 2022 Maize Genotype by Environment Prediction Competition data files

Label	Name of data file	File types (Extension)	Data repository and identifier
Data file 1	readme.txt	.txt	CyVerse (10.25739/tq5e-ak26) [3]
Data file 2	COMPETITION_DATA_README.docx	.docx	CyVerse (10.25739/tq5e-ak26) [3]
Data file 3	1_Training_Trait_Data_2014_2021.csv	.csv	CyVerse (10.25739/tq5e-ak26) [3]
Data file 4	2_Training_Meta_Data_2014_2021.csv	.csv	CyVerse (10.25739/tq5e-ak26) [3]
Data file 5	3_Training_Soil_Data_2015_2021.csv	.csv	CyVerse (10.25739/tq5e-ak26) [3]
Data file 6	4_Training_Weather_Data_2014_2021.csv	.csv	CyVerse (10.25739/tq5e-ak26) [3]
Data file 7	5_Genotype_Data_All_Years.vcf.zip	.vcf	CyVerse (10.25739/tq5e-ak26) [3]
Data file 8	6_Training_EC_Data_2014_2021.csv	.csv	CyVerse (10.25739/tq5e-ak26) [3]
Data file 9	All_hybrid_names_info.csv	.csv	CyVerse (10.25739/tq5e-ak26) [3]
Data file 10	GenoDataSources.txt	.txt	CyVerse (10.25739/tq5e-ak26) [3]
Data file 11	GenoDataSourcesWithUpdatedBioProject.txt	.txt	CyVerse (10.25739/tq5e-ak26) [3]
Data file 12	1_Submission_Template_2022.csv	.csv	CyVerse (10.25739/tq5e-ak26) [3]
Data file 13	2_Testing_Meta_Data_2022.csv	.csv	CyVerse (10.25739/tq5e-ak26) [3]
Data file 14	3_Testing_Soil_Data_2022.csv	.csv	CyVerse (10.25739/tq5e-ak26) [3]
Data file 15	4_Testing_Weather_Data_2022.csv	.csv	CyVerse (10.25739/tq5e-ak26) [3]
Data file 16	6_Testing_EC_Data_2022.csv	.csv	CyVerse (10.25739/tq5e-ak26) [3]
Data file 17	Test_Set_Observed_Values_ANSWER.csv	.csv	CyVerse (10.25739/tq5e-ak26) [3]

## Limitations

These datasets contain missing data. When working with large agricultural datasets, missing data is a common occurrence due to various factors such as data collection limitations, measurement errors, plot losses, and environmental events. The genotypic data provided contains hybrid information derived from inbred genotypic data, a common practice. However, depending on the study goals, this may pose limitations for specific types of analysis. In instances where precise GPS coordinates were not available for certain environments (i.e., a location in a particular year), field coordinates were estimated. Depending on the research objective, the unavailability of accurate GPS coordinates could impact the reliability of the results.

## Data Availability

The data described in this Data note can be freely and openly accessed on CyVerse under 10.25739/tq5e-ak26 [3]. Please see Table 1 for details and links to the data.

## Abbreviations

G2F: Genomes to Fields
GxE: Genotype by Environment
